# Updating a clinical prediction model for identifying monogenic diabetes to include both clinical features and biomarkers

**DOI:** 10.2337/dc25-1029

**Published:** 2026-04-01

**Authors:** Julieanne Knupp, Pedro Cardoso, Katherine G. Young, Timothy J. McDonald, Kashyap A. Patel, Kevin Colclough, Ewan R. Pearson, Angus G. Jones, Sophie Jones, Shivani Misra, Andrew T. Hattersley, Trevelyan J. McKinley, Beverley M. Shields

**Affiliations:** 1 University of Exeter Medical School. Address: Clinical and Biomedical Sciences, RILD Building, Royal Devon & Exeter Hospital, Barrack Road, Exeter EX2 5DW, UK; 2 Exeter Genomics Laboratory, Royal Devon University Healthcare NHS Foundation Trust, Exeter, United Kingdom; 3 University of Dundee. Address: Division of Population Health & Genomics, Ninewells Hospital and Medical School, University of Dundee, Dundee DD1 9SY, UK; 4 Department of Metabolism, Digestion & Reproduction, Imperial College London, London, UK

**Keywords:** Monogenic diabetes, Bayesian analysis, Clinical Decision Making, Biomarkers, Clinical Utility

## Abstract

**Objective:**

Selecting appropriate individuals for monogenic diabetes genetic testing is challenging. We aimed to develop a new probability calculator, integrating clinical features and biomarkers, to aid identification of monogenic diabetes.

**Research Design and Methods:**

We developed two prediction models (for early-insulin-treated, proxy for type 1 diabetes; and not-early-insulin-treated patients, proxy for type 2 diabetes) using a Bayesian recalibration mixture model approach. We used case-control data (monogenic diabetes=594, non-monogenic diabetes diabetes=597) for initial model development (clinical features only) and recalibrated to population-data (UNITED study, n=1,299) including biomarkers (C-peptide and islet-autoantibodies). We externally validated the calculator in an independent population-based cohort (n=1,025).

**Results:**

For early-insulin-treated individuals, the model incorporating biomarkers improved discrimination over using clinical features only (ROCAUC 0.98 [95%CrI 0.95–0.98] vs. 0.80 [95%CrI 0.71–0.82], p<0.001) or biomarkers alone (ROC AUC 0.96 [95% CI 0.95–0.97]). For not-early-insulin-treated participants, the calculator showed good discrimination (ROCAUC: 0.86 [95%CrI 0.85–0.88]). Both models calibrated well and showed good discrimination in external validation (0.98 and 0.92 for early- and not-early-insulin-treated individuals, respectively). Using a ≥5% probability threshold to guide testing results in positive test rates for monogenic diabetes of 16–19%.

**Conclusions:**

We developed an updated monogenic diabetes probability calculator that integrates both clinical features and biomarkers, providing greater discrimination than using clinical features or biomarkers alone and providing appropriate measures for selecting individuals for monogenic diabetes diagnostic testing. This is now available as an online calculator and has immediate clinical utility for White European individuals diagnosed with diabetes ≤35 years.

## Introduction

Monogenic diabetes (MD) is a genetic form of diabetes estimated to account for 1-5% of young-onset diabetes(1,2). It is often misdiagnosed as type 1 or early-onset type 2 diabetes(1). Correctly diagnosing MD has important implications for a patient’s treatment(3), the management of their condition, and the likelihood of family members being affected(4–6). MD is diagnosed via expensive genetic testing, so, for cost-effective screening, testing needs to be targeted to those most likely to have it.

Biomarkers can be helpful in differentiating MD from type 1 diabetes in insulin-treated individuals. Islet autoantibodies (e.g. Glutamic acid decarboxylase - GAD, and Islet Antigen 2 - IA2) are markers of beta cell autoimmunity associated with type 1 diabetes but rarely seen in MD(7). C-peptide is a marker of endogenous insulin secretion, so people with type 1 diabetes generate low amounts of C-peptide, whereas in MD, C-peptide production is retained(8). Islet autoantibodies are most accurate when measured near diagnosis(9), as they tend to degrade later in type 1 diabetes progression, whereas C-peptide is most useful 3-5 years after diagnosis, as it may be elevated during the honeymoon phase(8). Whilst low C-peptide and positive islet autoantibodies have been proposed as useful biomarkers for ruling out individuals for genetic testing, these are only helpful in those who are insulin-treated, as they have far less benefit discriminating MD from type 2 diabetes(10). Using these biomarkers alone results in a large proportion of individuals eligible for testing that do not have the condition, resulting in correspondingly low positive test rates (PPV=8-10%(11,12)).

Clinical features have been crucial in discriminating MD from type 1 and type 2 diabetes. Family history of diabetes, non-obesity and lack of insulin treatment have traditionally been used to select individuals for genetic testing(6,13,14). A more sophisticated tool for determining individuals likely to have the most common forms of MD is the Maturity Onset Diabetes of the Young (MODY) probability calculator(15). The online calculator (available at https://www.diabetesgenes.org/exeter-diabetes-app/) uses routine clinical features to generate probability estimates, built on case-enriched data. These probabilities are then adjusted to the MODY prevalence in the young-onset diabetes (diagnosed <35 years) population. This provides clinicians with an easily interpretable measure that can help guide decisions regarding genetic testing. It features in international guidelines(3,16,17) and is part of NHS England’s National Genomic Testing Criteria(18).

Whilst clinicians use both the “MODY calculator” and biomarker tests to aid decisions around MD testing, there has not yet been a way of combining this information meaningfully. Therefore, probabilities for those who are C-peptide positive (i.e. produce sufficient endogenous insulin) or islet autoantibody negative may be underestimated. Combining these biomarkers with clinical features in the MODY calculator would have clear advantages: improving the positive test rate in insulin-treated individuals where the performance of the current model is weaker(15), and enabling appropriate determination of a patient’s likelihood of MD when using combined information. Additional improvements to the MODY calculator are warranted. The MODY calculator only provides twenty set probabilities, rather than continuous probabilities, and it was developed based on only *GCK, HNF1A* and *HNF4A-*MODY cases; its ability to detect MD with different genetic aetiologies is unknown.

We aimed to 1) develop a new monogenic diabetes calculator that integrates clinical features and biomarkers for early-insulin-treated individuals, calibrated to the young-onset diabetes population to provide continuous probabilities for MD, and for a wider range of genetic aetiologies; 2) assess the calculator’s calibration and discrimination performance in the young-onset diabetes population for early- and not-early-insulin-treated individuals; and 3) assess the calculator’s clinical utility using different probability thresholds for testing.

## Research design and methods

Models were developed on people diagnosed with diabetes aged 1-35y, excluding those likely to have neonatal diabetes (<1y) and those >35y, where type 2 diabetes predominates and MD is rarer. Since features discriminating MD from type 1 and young-onset type 2 diabetes differ substantially(15), we developed two models: one for use in early-insulin-treated individuals (insulin-treated within six months of diagnosis; a proxy for type 1 diabetes) and one for use in not-early-insulin-treated individuals (no insulin treatment or insulin-treated >6 months from diagnosis; a proxy for type 2 diabetes).

**Figure 1:**
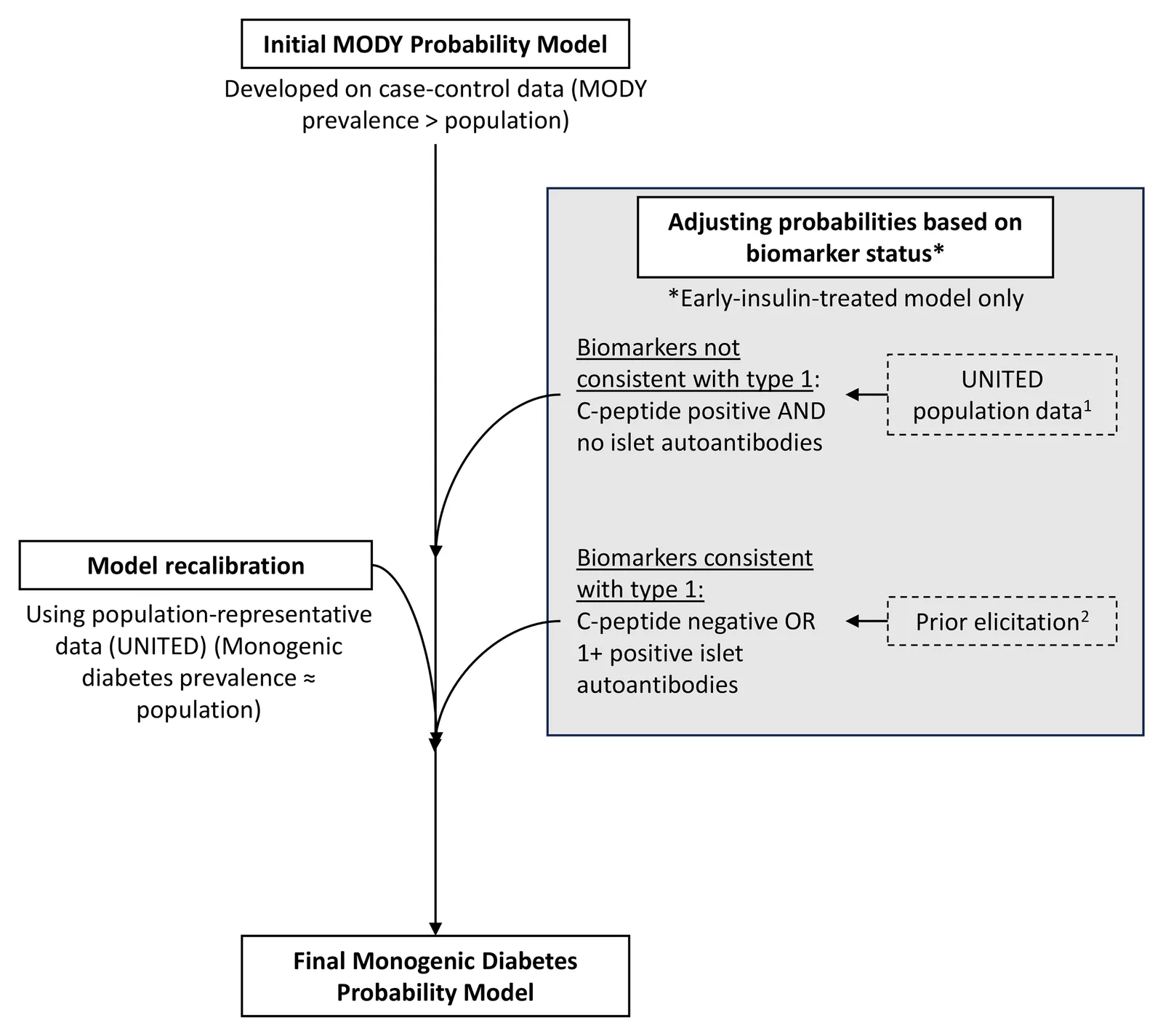
Flow diagram describing the model development and recalibration approach used to build the Monogenic Diabetes Probability Calculator. Initial monogenic diabetes probabilities are developed on case-control data, for early- and not-early-insulin-treated individuals (39% and 57% monogenic diabetes cases) respectively and recalibrated to population-representative data (0.9% and 18.4% monogenic diabetes cases respectively). For the early-insulin-treated model only (right; grey box), biomarker status information, based on C-peptide status (positive: C-peptide ≥200pmol/L or UCPCR ≥0.2nmol/mmol or fasting C-peptide ≥80pmol/L; negative: C-peptide <200pmol/L or UCPCR <0.2nmol/mmol or fasting C-peptide <80pmol/L) and presence of islet autoantibodies, is integrated into the model. Dashed boxes represent references of methods informing how biomarkers are incorporated into the early-insulin-treated model. 1. Shields *et al* (20). 2. Cardoso *et al*. (19).

Since MD is relatively rare, appropriate datasets are scarce, so we combined data from two sources to maximise available information and improve model robustness: 1) a case-control training dataset to robustly estimate the odds ratios for MD using clinical features, and 2) a population-representative dataset to recalibrate the training model probabilities to reflect prevalence of MD in a young-onset diabetes population(19) (Figure 1).

For early-insulin-treated individuals, we wanted to incorporate additional biomarker (C-peptide and islet autoantibody) information, which were not available in the training data but were available in the population dataset. We used a Bayesian recalibration mixture model approach to amalgamate these different sources of information(19) (Figure 1 and Statistical analysis section).

We performed internal validation in the population-representative dataset, tested the overall calibration (appropriateness of probabilities when applied to a whole population) using GP records, and externally validated the calculator using additional population data with MD testing.

### Subject populations and data sources

#### Training data (enriched for monogenic diabetes)

We used the same case-control data that was used to develop the original MODY prediction models(15). MD cases had confirmed diagnoses through the Exeter Genomics laboratory and controls were recruited through local research cohorts(15). All participants were White European and diagnosed with diabetes aged 1-35y inclusive. This dataset includes 455 early-insulin-treated participants (177 MD, 278 non-MD) and 736 not-early-insulin-treated participants (417 MD, 319 non-MD).

#### Population-representative recalibration data

We used the UNITED study, a population-representative cohort of young-onset diabetes(20), to recalibrate the predictions to provide population-representative probabilities(19). The UNITED study recruited 62% of all individuals with diabetes diagnosed aged 1-30y in two regions of the UK (Exeter and Tayside, n=1,418)(20). This complete-case dataset (n=1,299), post-exclusions, included 1,174 early-insulin-treated participants (10 MD cases; 0.9% prevalence) and 125 not-early-insulin-treated participants (23 MD cases; 18.4% prevalence) (Supplementary Figure 1).

#### Calibration in GP records

A young-onset diabetes population-representative cohort was defined from the CPRD Aurum dataset(21) (UK primary care electronic healthcare records data). We analysed data from 67,827 individuals with any diabetes code from 2004 onwards, aged ≥18 years on 1^st^ January 2024, diagnosed aged 1-35y with a current diagnosis of type 1 or type 2 diabetes and all relevant variables (Supplementary Figure 2). Patient characteristics were defined based on values available closest and prior to the index date (1^st^ January 2024). MD is poorly recorded in primary care records and genetic testing rarely performed so MD status was considered unknown for all patients. This dataset was used to estimate overall calibration performance only (i.e. appropriateness of model probabilities when applied to a whole population) by comparing the mean predicted model probabilities to the expected proportion of MD cases estimated by previous literature. It could not be used for full external validation due to the unknown MD status in most cases.

##### Categorisation of diabetes in CPRD

Due to missing or unreliable time-to-insulin information, validation of the early-insulin-treated model was done in individuals with any of the following traits: currently on insulin only (last 12 months); had a type 1 diabetes code, diagnosed <10y with an HbA1c >58 mmol/mol (7.5%); or diagnosed with type 1 diabetes <25y, and currently treated with metformin and insulin only(n=35,150; 51.8%). The not-early-insulin-treated model was applied to all other individuals (n=32,677; 48.2%) (Supplementary Figure 2).

#### External validation data

To maximise numbers and validate the calculator across a similar age range to the development cohorts, we combined data from White European individuals from two population-representative studies:

1. The UNITED paediatric cohort(22): participants with diabetes aged <20y in a dataset of Southwest UK paediatric individuals recruited separately from the UNITED study, following the same study protocol (n=425).
2. The MY DIABETES study(23,24): participants (predominantly adults) diagnosed <30y. For consistency, we analysed the subset of individuals of White European ethnicity (n=600).

Together the external validation dataset consisted of 940 early-insulin-treated participants (5 MD cases; 0.5% prevalence) and 85 not-early-insulin-treated participants (15 MD cases; 17.6% prevalence) (Supplementary Figure 3).

## Model predictors

### Clinical features

All datasets comprise of the following variables which were collected at study recruitment (index date for CPRD): sex, age-at-diagnosis (years), current age (years), most recent BMI (kg/m^2^), parent history of diabetes, most recent HbA_1c_ (%), and current treatment (insulin, other glucose-lowering treatment or none).

### Biomarkers

Biomarker data was not available for the training dataset or CPRD. In both UNITED cohorts, C-peptide (UCPCR) was available on all early-insulin-treated participants (UNITED: n=1,174 (median duration 13.7y [IQR:5.7, 22.9]); UNITED paediatric: n=409 (median duration 4.0y [1.6, 7.3])). GAD and IA2 islet autoantibodies were available for all participants with retained C-peptide (UCPCR ≥0.2nmol/mmol; UNITED: n=250; UNITED paediatric: n=119). In MY DIABETES, plasma C-peptide was available on all but six early-insulin-treated participants (n=525; median duration 18.8y [IQR 9.0, 32.8]). GAD and IA2 islet autoantibodies were available for all participants with detectable C-peptide (assay limit >27pmol/L) (n=123). Individuals were considered positive for islet autoantibodies if their results were >99th centile (GAD: 64WHOunits/ml; IA2: 15WHOunits/ml)(7). Biomarker status was coded as a binary variable:

- Biomarkers consistent with type 1: C-peptide negative (UCPCR <0.2nmol/mmol; unfasted plasma C-peptide <200pmol/L; fasted plasma C-peptide <80pmol/L) or having one or more positive islet autoantibody or
- Biomarkers not consistent with type 1: C-peptide positive (UCPCR ≥0.2nmol/mmol; unfasted plasma C-peptide ≥200pmol/L; fasted plasma C-peptide ≥80pmol/L) and islet autoantibody negative.

### Model outcome

All MD testing for the cohorts in this study was carried out at the Exeter Genomic Laboratory using NGS gene panels. We defined MD as having a genetic diagnosis of higher penetrance MD genes *GCK, HNF1A, HNF4A, HNF1B, ABCC8, KCNJ11* and *INS*. Other syndromic monogenic subtypes in these datasets were excluded (*MIDD, TRMT10A, MFP, WFS1, INSR, LMNA, PPARG*) as referral for testing may be based on other features (UNITED recalibration: n=11; external validation: n=3, Supplementary Figures 1&3). All cases in the training dataset had genetically confirmed MD (based on *GCK, HNF1A* or *HNF4A* genes). Controls were assumed not to have MD(15).

In both UNITED cohorts, genetically confirmed MD status was available for all not-early-insulin-treated participants, and early-insulin-treated participants with biomarkers not consistent with type 1 diabetes (retained C-peptide and no positive islet autoantibodies; UNITED: n=191; UNITED paediatric: n=37), in addition to participants with previously diagnosed MD (Supplementary Figure 1). For all other participants (i.e. early-insulin-treated with biomarkers consistent with type 1 diabetes) genetic testing was not available as they were assumed to have type 1 diabetes(7,20). Uncertainty relating to this was built into the statistical model(19) (Figure 1, Supplementary Figure 5).

In MY DIABETES, genetically confirmed MD status was available for all participants with biomarkers not consistent with type 1 diabetes (n=71).

### Statistical analysis

Statistical analysis was carried out in R (v4.3.2)(25) using primarily *nimble* (v1.0.1)(26) and *tidyverse* (v2.0.0)(27) packages. TRIPOD+AI Guidelines and checklists were followed and completed(28) (Supplementary Figure 6).

#### Model development

The early-insulin-treated model was developed using a “Recalibration mixture” method(29) in a Bayesian hierarchical shrinkage mixture model(19) (Figure 1). This approach uses case-control training data to build an initial model using the larger numbers of MD cases to get more precise estimates of the log-odds ratios for the clinical features, before recalibrating the model probabilities using the population data (UNITED study) to ensure the resulting predictions are appropriately calibrated for the young-onset population-of-interest. The early-insulin-treated model can function without the requirement of biomarker information (i.e. on clinical features alone, Supplementary Figure 5). The not-early-insulin-treated model was similarly developed using the Bayesian “Recalibration” method(29) in a Bayesian setting(19), but without the integration of the biomarkers step. Credible intervals (95%) are reported as CrI.

#### Discriminative ability of the models and internal calibration

Receiver Operating Characteristic (ROC) curve analysis(30) was performed for the early-(clinical features alone, and clinical features and biomarkers) and not-early-insulin-treated models in UNITED, and the Area Under the Curve (AUC) estimated. Overall internal calibration was assessed by examining mean model probabilities for each model, within MD and non-MD participants, and producing calibration plots (i.e. comparing predicted probabilities against observed proportions with MD).

#### Calibration estimation in external population data

MD probabilities were calculated in CPRD for both early- and not-early-insulin-treated individuals. These probabilities were used to assess the mean MD probability within a young-onset diabetes population-representative dataset and to check whether this was in line with the expected MD prevalence in a young-onset diabetes population (3.6% in UNITED). Due to the low proportion of biomarker information in CPRD (0.1%), we assessed the calibration for the clinical features only models.

#### Assessment of clinical utility

To ascertain the clinical utility of the early- and not-early-insulin-treated models when combined in an overall monogenic diabetes calculator, we determined the proportions of MD identified and missed if using probability thresholds of ≥5%, ≥10%, ≥20%, or ≥30% to guide genetic testing. These potential genetic testing thresholds were examined in UNITED and the external validation cohort. Additional thresholds were tested and reported in the Supplementary Materials. The monogenic diabetes calculator was compared to a biomarker screening pathway, traditional MODY criteria(20), NHS genomic testing guidelines(18), and the previous MODY calculator(15).

#### External validation

Both models were validated in the combined independent paediatric UNITED and MY DIABETES cohorts, and performance assessed using ROC curves, calibration plots and different probability thresholds.

#### Sensitivity analysis

The early-insulin-treated model was further validated in paediatric subgroups of the external validation: in those diagnosed <18y, and those recruited <18y.

## Results

### Participant characteristics

Participant characteristics for the training, recalibration, CPRD, and external validation datasets are shown in Supplementary Tables 1–4.

### Model development

The models calibrated well, with the mean probabilities of both models in UNITED (0.9% and 18.4%, for early- and not-early-insulin-treated UNITED participants, respectively) consistent with the raw proportions seen in UNITED (0.9% and 18.4%) (Supplementary Figure 7). The mean MD probability in UNITED across participants was 2.6%. Bootstrap internal validation proved stable across both models.

### Calculator with biomarkers and clinical features has improved discrimination over clinical features alone

**Figure 2:**
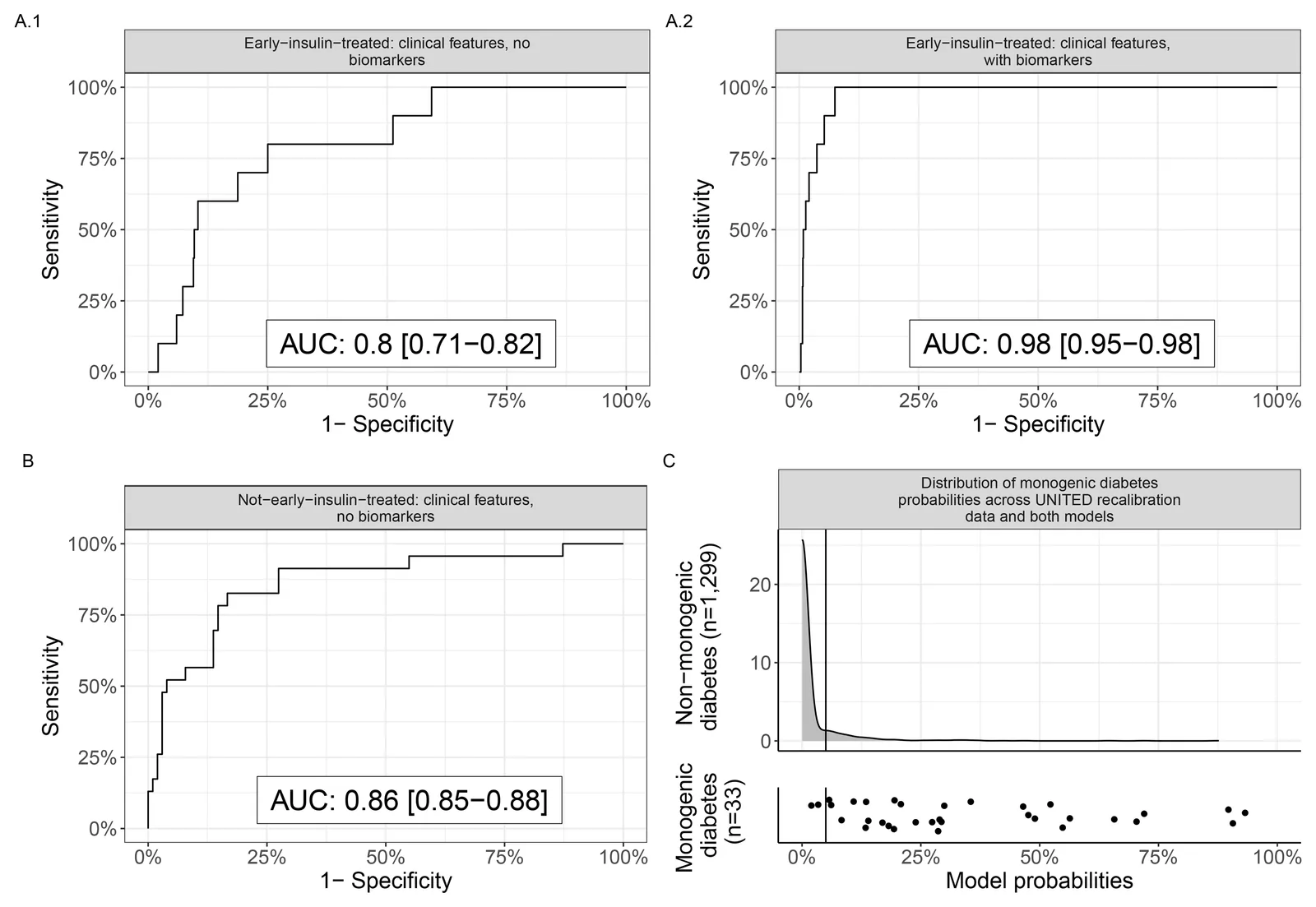
ROC curves showing the discriminatory ability of A) early-insulin-treated diabetes vs. monogenic diabetes, for a model using clinical features alone (A.1), and clinical features and biomarkers (A.2); and B) not-early-insulin-treated diabetes vs. monogenic diabetes in UNITED population-representative recalibration data. Black lines denote the ROC curve of the mean probabilities. Square brackets denote 95% Credible Intervals around the ROCAUC. C) shows the distribution of monogenic diabetes Calculator probabilities in combined UNITED population-representative recalibration data split by non-monogenic diabetes participants (density curve) and monogenic diabetes cases (dots). Vertical black line represents the monogenic diabetes probability of 5%, suggested as a testing threshold.

For early-insulin-treated individuals, the model combining clinical features and biomarkers showed excellent discrimination in UNITED. The combined model had improved discrimination over using clinical features alone (ROCAUC 0.98 [95%CrI 0.95–0.98] vs. 0.80 [95%CrI 0.71–0.82], p<0.001; Figure 2A) and over biomarkers alone (ROCAUC 0.96 [95% CI 0.95–0.97], p=0.046; Supplementary Figure 8).

The model with clinical features and biomarkers increased the probabilities in participants with MD (mean probability of 14.0% using the model with biomarkers vs. 2.1% with clinical features alone), with a marginal decrease in probabilities in those without MD (mean probability 0.8% vs. 0.9% for model with biomarkers vs. with clinical features alone; Supplementary Figure 9A).

For not-early-insulin-treated participants in UNITED, the calculator showed good discrimination (ROCAUC: 0.86 [95%CrI 0.85-0.88]; Figure 2B). The probabilities in the not-early-insulin-treated model were generally higher than in the early-insulin-treated model, reflecting the higher prevalence of MD in this group. Those with MD had a predicted mean probability of 44.5%, compared to 12.5% in those without MD (Supplementary Figure 9B).

### The monogenic diabetes calculator probabilities are consistent with expected population prevalence

When assessing the calibration of the models in CPRD, the predicted probabilities were in line with the young-onset diabetes population prevalence (Supplementary Figure 10), with a mean probability of 3.8%, consistent with the UNITED population estimate (3.6% in patients diagnosed <30y(20)).

### Clinical utility

Table 1 shows summary information of using different probability thresholds for indicating genetic testing in practice. Using a ≥5% threshold for all participants in UNITED, would result in 12.9% (n=163/1,299) being eligible for genetic testing. Of these, 19.0% (n=31/163) had MD (a positive test rate of ~1 in 5). This threshold missed two of 33 cases (6.1%) (Figure 2C).

Using lower thresholds yielded a higher percentage of the population being eligible for testing, and fewer MD cases being missed, but a lower positive test rate (and vice versa for higher probability thresholds) (Supplementary Table 5).

Supplementary Table 6 compares the use of the monogenic diabetes calculator to alternative screening strategies. If genetic testing were limited based on biomarker information alone (i.e. test all C-peptide positive and islet autoantibody negative), a greater number of individuals would be eligible for genetic testing (17.2%), with a lower positive test rate (14.8%), and no MD cases missed. Conversely, if traditional MODY clinical criteria were used, a much smaller number of individuals would be eligible for genetic testing (2.8%), with a greater positive test rate (40.5%), however 54.5% of cases would be missed. Compared with the original MODY calculator (no biomarkers), ≥5% probability on the updated monogenic diabetes calculator would result in an improved positive test rate, although performance was similar when using ≥5% on the original MODY calculator in conjunction with biomarkers. The 5% threshold has similar performance to the NHS genomic testing criteria.

### Model validation

**Figure 3:**
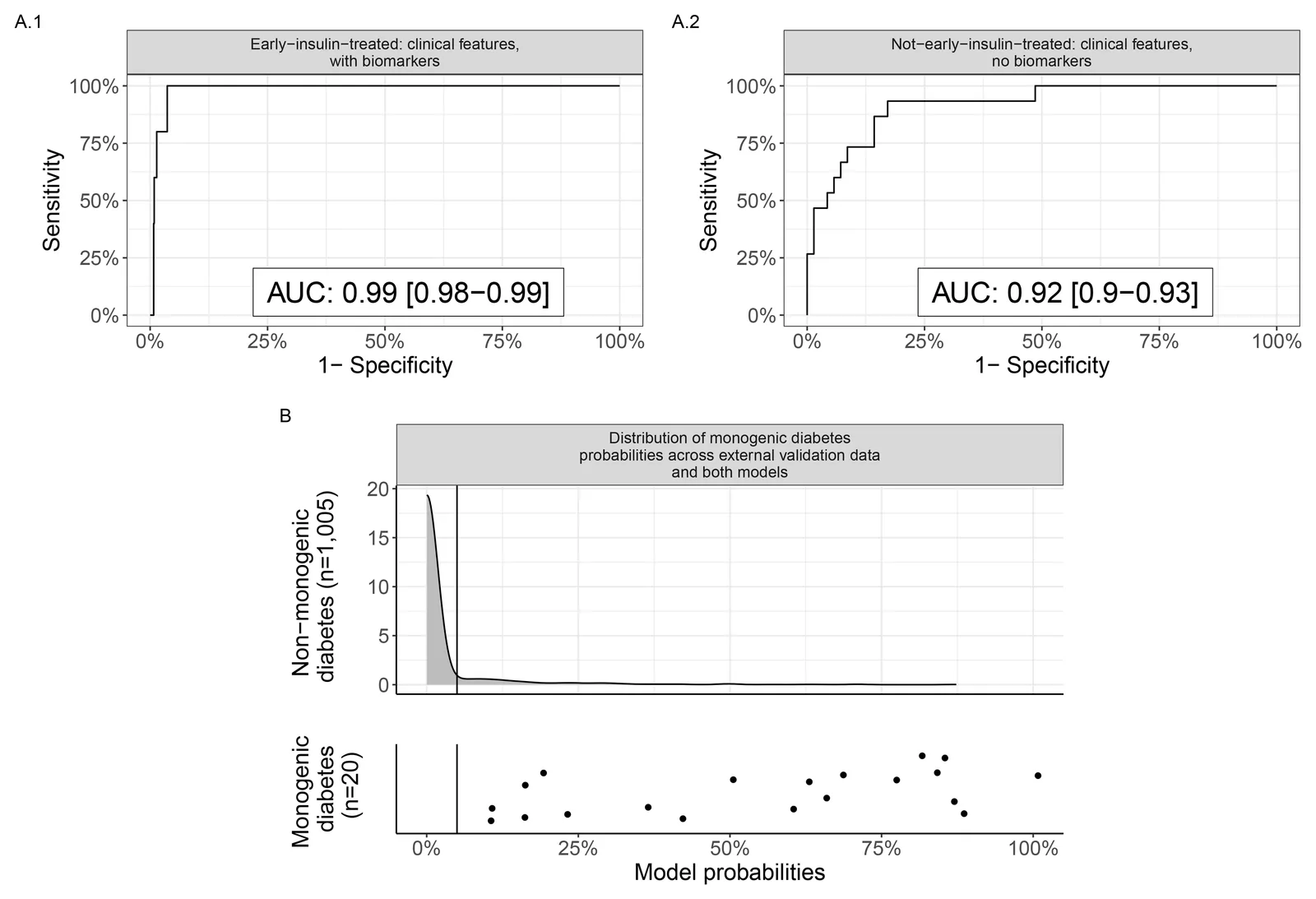
Discrimination and distribution of the Monogenic Diabetes Probability Calculator in the external validation dataset. Receiver Operating Characteristic curves for A.1) early-insulin-treated and A.2) not-early-insulin-treated models show Areas Under the Curve of 0.98 [95%CrI 0.98–0.98] and 0.92 [95%CrI 0.9–0.93] respectively. Square brackets denote 95% Credible Intervals (CrI) around the ROCAUC. B) shows the distribution of monogenic diabetes Calculator probabilities in the combined external validation, split by non-monogenic diabetes participants (density curve) and monogenic diabetes cases (dots). Vertical black line represents the monogenic diabetes probability of 5%, suggested as a testing threshold.

The early-insulin-treated model demonstrated a ROCAUC of 0.98 [95%CrI 0.98-0.98] in the external validation dataset (Figure 3A.1). MD cases (n=5; 0.5%) had a much higher predicted mean probability compared to those without MD (16.3% vs. 0.68%; p<0.0001).

The not-early-insulin-treated model had a ROCAUC of 0.92 [95%CrI 0.90-0.93] (Figure 3A.2). MD cases (n=15; 17.6%) had a much higher predicted mean probability compared to those without MD (66.7% vs. 22.3%, p<0.0001).

Using a genetic testing threshold of ≥5% for the monogenic diabetes calculator across all participants, resulted in a positive test rate of 16.1% (Supplementary Table 7), with no MD cases missed (Figure 3B).

Calibration in the early-insulin-treated model was reasonable (Supplementary Figure 11A). There was some suggestion of slight overestimation in the not-early-insulin-treated model (Supplementary Figure 11B), but confidence intervals overlapped with the calibration line and the calibration-in-the-large was zero, indicating acceptable calibration (i.e. predicted probabilities line up well with observed proportions with MD).

### Sensitivity analysis

The early-insulin-treated model showed great discrimination in the paediatric subgroups of the external validation dataset (n=718 (3 MD) diagnosed <18y; n=438 (2 MD) recruited <18y). When using a ≥5% genetic testing threshold, all MD cases would be picked up, and only 3.5 and 5% of the respective paediatric populations would be eligible for testing (Supplementary Table 8).

### Online calculator

The model equations have been turned into an online prototype calculator available at https://pm-cardoso.shinyapps.io/MODY_calculator/. The online prototype calculator returns the probability of MD based on clinical features and biomarkers using the equations from the statistical models presented in this paper. Model coefficients for the online prototype calculator are shown in Supplementary Table 9.

## Conclusions

We have developed a monogenic diabetes probability calculator for use in White European populations that for the first time integrates clinical features and biomarkers and yields better discrimination over using clinical features or biomarkers alone for detecting MD in early-insulin-treated individuals. Two models were developed that demonstrate excellent discrimination in both early-(type 1-like phenotype) and not-early-insulin-treated (type 2-like phenotype) individuals. Integration of biomarkers improved the discrimination in early-insulin-treated individuals from ROCAUC 0.80 to 0.98. Models performed well in external validation (ROCAUC: 0.98 and 0.92 for early- and not-early-insulin-treated individuals) and probabilities were appropriately calibrated for the population (mean probability in UK CPRD population data = 3.8%, similar to the expected proportion based on prevalence studies). These new validated models are now available as a prototype calculator, providing probabilities adjusted for biomarker information.

The primary strength of this work is that the monogenic diabetes probability calculator combines information from routinely available clinical features and biomarkers, providing improved discrimination over using features in isolation, and presents the outputs in a user-friendly format to aid clinical decisions. By using the Bayesian recalibration approach, we were able to combine information from multiple sources to overcome some of the problems with developing prediction models in rare disease(19). In contrast to the previous calculator, we have shown this new calculator can predict MD defined by broader range of genes, beyond that in the original MODY calculator(15), allowing for a unified prediction when testing MD using a monogenic NGS panel(31). A major improvement is that this calculator now provides population-representative probabilities on a continuous scale, allowing the clinician to place their patient reliably within the context of the young-onset diabetes population.

By using population data, we have been able to assess for the first time the calibration of the model probabilities in independent external validation datasets, and primary care records. We found that the mean probability from the calculator reflected the estimated prevalence of MD, suggesting the probabilities were overall appropriate for young-onset diabetes (both type 1 and type 2 phenotypes) in a White European population. The monogenic diabetes calculator has improved performance and greater positive test rate than using the biomarker screening pathway alone and ensures that nearly all MD cases are picked up in comparison to using traditional clinical criteria(20). Sensitivity analysis demonstrated that it is effective in paediatric populations.

We acknowledge that a great limitation of our calculator is that it remains only for use in individuals of White European ancestry. Recent work has suggested the original MODY calculator may overestimate probabilities in populations with higher prevalence of young-onset type 2 diabetes(32,33). This likely reflects the fact that type 2 is diagnosed at a younger age and slimmer BMI, meaning key criteria in the model are less discriminatory. Unfortunately, the datasets utilised in our study, contained very low numbers of individuals of non-White European ancestry. Determining larger datasets of diverse ethnicities to robustly test and appropriately adjust the models is the focus of ongoing work.

The models were developed using prevalent datasets comprising patients many years after diagnosis and a key criterion for the calculator is time-to-insulin within six months, meaning it cannot be used at time of diagnosis. Furthermore, C-peptide may be raised close to diagnosis in type 1 diabetes due to the honeymoon period. For paediatric patients, an alternative calculator has been developed for use at diagnosis that places greater emphasis on islet autoantibody results, and this would be preferable to use in this situation(34).

Participants who were early-insulin-treated with low C-peptide or positive islet autoantibodies (consistent with a diagnosis of type 1 diabetes) were not genetically tested in the datasets utilised due to cost, but the probability of MD in this group is extremely low (estimated to be 0.02%(7,19)), and this uncertainty was accounted for in the models.

Determining the most appropriate testing thresholds of our monogenic diabetes probability calculator will be a decision for each testing body or laboratory. The optimum threshold will be a balance between the cost of testing, the likelihood of a positive result and the patient benefit. Limiting testing to a higher probability threshold may result in fewer tests but more MD individuals being missed, whereas using a lower probability threshold will result in more cases being detected, but greater expense due to more testing. As an example, using a probability threshold of ≥5% would yield a similar performance to the current NHS England genomic testing criteria, but with fewer missed MD cases. This probability threshold provides a positive test rate of 16.1–19.0%, with 12.1–12.9% of the young-onset diabetes population being eligible for testing.

We have developed a monogenic diabetes probability calculator that integrates clinical features and biomarkers and is appropriate for screening in the general young-onset diabetes population for a wide range of MD genes. This monogenic diabetes probability calculator provides greater discrimination than using clinical features alone for early-insulin-treated individuals and provides appropriate measures for selecting individuals for diagnostic testing.

## Supplementary Material

Supplementary

## Figures and Tables

**Table 1:** Assessment of potential testing thresholds of the Monogenic Diabetes Probability Calculator in the combined UNITED dataset. Table denotes the proportion of the population with a MD probability greater than or equal to the defined monogenic diabetes testing thresholds, and the corresponding percentage of monogenic diabetes cases missed (proportion of monogenic diabetes cases not detected if testing all above this threshold), positive test rate (PPV, proportion of those tested being positive for monogenic diabetes if testing all above this threshold), negative test rate (NPV, proportion below the threshold that have non-monogenic diabetes), sensitivity (proportion of all monogenic diabetes cases that are above the threshold) and specificity (proportion of all non-monogenic diabetes cases that are below the threshold).

UNITED (n=1,299; 33 monogenic diabetes cases within this group)						
Threshold	% individuals tested at threshold	% monogenic diabetes cases missed	% positive test rate (PPV)	% negative test rate (NPV)	Sensitivity	Specificity
≥5%	12.5% (163/1,299)	6.1% (2/33)	19.0% (31/163)	99.8% (1,134/1,136)	93.9% (31/33)	89.6% (1,134/1,266)
≥10%	7.3% (95/1,299)	15.2% (5/33)	29.5% (28/95)	99.6% (1,199/1,204)	84.8% (28/33)	94.7% (1,199/1,266)
≥20%	3.4% (44/1,299)	39.4% (13/33)	45.5% (20/44)	99.0% (1,209/1,255)	60.6% (20/33)	98.1% (1,209/1,266)
≥30%	2.2% (28/1,299)	60.6% (20/33)	46.4% (13/28)	98.4% (1,251/1,271)	39.4% (13/33)	98.8% (1,251/1,266)

## Data Availability

Training and UNITED data are available through application to the Genetic Beta Cell Research Bank https://www.diabetesgenes.org/current-research/genetic-beta-cell-research-bank/ and the Peninsula Research Bank https://exetercrfnihr.org/about/exeter-10000-prb/. CPRD Aurum data (eRAP: 23_003615)(21) can be requested via https://www.cprd.com/. The MY DIABETES study is subject to data governance requirements. Access to the data is restricted and may be granted to qualified researchers upon reasonable request subject to approval by the relevant data governance and ethics committees (email smisra@imperial.ac.uk). All statistical code and CPRD cohort scripts can be found at https://github.com/Exeter-Diabetes/MODY-calculator-clinical-paper.

## Twitter summary

We have developed a monogenic diabetes calculator that integrates clinical features, C-peptide and islet autoantibody results to help prioritise individuals who may benefit from genetic testing.

## Article Highlights

- **Why did we undertake this study?** Monogenic diabetes is a form of diabetes that is challenging to identify. Approaches are needed to help determine which patients should receive diagnostic genetic testing.
- **What is the specific question(s) we wanted to answer?** We aimed to develop a monogenic diabetes calculator integrating both clinical features and biomarkers.
- **What did we find?** We developed and validated prediction models for monogenic diabetes demonstrating good discrimination and calibration. The integration of biomarkers greatly improved the discrimination over clinical features alone.
- **What are the implications of our findings?** The validated models, integrating both clinical features and biomarkers, are now available as a prototype online calculator. This represents a step-change over current clinical approaches for selecting appropriate patients for diagnostic genetic testing for monogenic diabetes.
