# Supplementary material for: Updating a clinical prediction model for identifying monogenic diabetes to include both clinical features and biomarkers
Source: Diabetes Care. Author manuscript; Available in PMC 2026 Oct 30. (PMC7619657; doi:10.2337/dc25-1029)
Supplement: Supplementary file 1 [file EMS219681-supplement-Supplementary_.docx]

Supplementary Materials

**Supplementary Figure 1: Flow diagram of individuals used from the population-representative UNITED study, used to recalibrate models**. Depicted is the biomarker screening pathway utilised as part of the study for individuals with no known genetic cause for their diabetes; for early- (blue) and not-early-insulin-treated individuals (green). Adapted from Shields *et al.* 2017. Biomarker testing refers to UCPCR and islet autoantibody testing. *Biomarkers consistent with type 1 refers to UCPCR <0.2nmol/mmol or one or more positive islet autoantibodies, whereas biomarkers not consistent with type 1 refers to UCPCR ≥0.2nmol/mmol and no positive islet autoantibodies.

**Supplementary Figure 2: CPRD Aurum June 2024 release with 2,727,999 unique individuals with a diabetes-related medcode between 01/01/2004 and 30/06/2024** (download date). This was filtered to adults that were registered at a GP on 1^st^ January 2024, in addition to other basic quality control steps (which can be found at https://github.com/Exeter-Diabetes/CPRD-Pedro-monogenic diabetes). Due to missing/unreliable time to insulin information, type 1 individuals are defined by having any of the following: currently on insulin only (within the last 12 months); had a type 1 diabetes code, was diagnosed under the age of 10 years and had an HbA1c greater than 58 mmol/mol; or was diagnosed with type 1 diabetes under the age of 25 years of age and is currently treated with metformin and insulin only. The not-early-insulin-treated model was applied to all other individuals (as a proxy for type 2 diabetes).

**Supplementary Figure 3: Simplified diagram depicting the construction of the external validation datasets used for the early- (blue) and not-early-insulin-treated (green) models.** This is formed from the MY DIABETES study (n=600) and the UNITED paediatric study (n=425).

**Supplementary Figure 4: Breakdown of monogenic diabetes genes in training (case-control data, enriched for monogenic diabetes cases), recalibration (UNITED study) and external validation (combination of UNITED paediatric and MY DIABETES studies) data.** Early-insulin-treated monogenic diabetes cases are displayed in coral-red and not-early-insulin-treated monogenic diabetes cases in turquoise blue.

**Supplementary Figure 5: Flow diagram explaining the difference between the early-insulin-treated model with clinical features and biomarkers and the model with clinical features only.** The top two arrows denote the model with clinical features and biomarkers, which uses whether the biomarker information is consistent with type 1 (middle) or not (top) to separate how the probabilities of monogenic diabetes are derived. The bottom arrow explains the model with clinical features only, which uses the clinical features to first calculate the predicted probability of having biomarkers consistent with type 1 and not consistent with type 1, before secondly using that information in a combined form with the ways of estimating the predicted probability of monogenic diabetes described above this. Blue shaded box indicates model format when no biomarker information is known (“clinical features, no biomarkers” model). Green shaded boxes indicate predicted probability of monogenic diabetes for those with biomarkers consistent with type 1, as estimated through prior elicitation. Orange shaded boxes indicate model form when biomarkers are not consistent with type 1 (“clinical features, with biomarkers” model). ^1^ Cardoso *et al.* 2024. *Based on model equations in Supplementary Table 9. **Probability of having biomarkers consistent with type 1 is calculated based on a model using all clinical features, with splines on BMI, age of diagnosis and current age. ***Calculated as 1-Predicted probability of having biomarkers consistent with type 1.

**Supplementary Figure 6: TRIPOD+AI Checklist**

**Supplementary Table 1: Participant characteristics of training case-control data for model relevant variables, enriched for monogenic diabetes cases^a^.** Values are displayed as median [interquartile range] for numeric variables. Otherwise, categorical variables are displayed as n (%).

|  |  | Early-insulin-treated (n=455) | | Not-early-insulin-treated (n=736) | |
| --- | --- | --- | --- | --- | --- |
|  |  | **Monogenic diabetes** | **Non-monogenic diabetes** | **Monogenic diabetes** | **Non-monogenic diabetes** |
| n |  | **177** | **278** | **417** | **319** |
| Gene | **GCK** | 45 (25.4%) | 0 (0%) | 198 (47.5%) | 0 (0%) |
|  | **HNF1A** | 103 (58.2%) | 0 (0%) | 193 (46.3%) | 0 (0%) |
|  | **HNF4A** | 29 (16.4%) | 0 (0%) | 26 (6.2%) | 0 (0%) |
| Age at diagnosis (years) |  | 17 [14,24] | 13 [7,22] | 17 [13,22] | 32 [29,34] |
| Age at recruitment (years) |  | 26 [16,34] | 37 [11.3,51] | 26 [16,36] | 42 [36.5,50] |
| Duration of diabetes (years) |  | 4 [1,11] | 17 [0,35] | 4 [1,15] | 10 [5,20] |
| Sex | Female | 133 (75.1%) | 147 (52.9%) | 278 (66.7%) | 160 (50.2%) |
|  | Male | 44 (24.9%) | 131 (47.1%) | 139 (33.3%) | 159 (49.8%) |
| BMI (kg/m^2^) |  | 23 [21,26] | 24 [18,28] | 23 [21,26] | 33 [28,38] |
| HbA1c (mmol/mol) |  | 49 [43, 62] | 66 [61,84] | 48 [43,54] | 66 [56,78] |
| HbA1c (%) |  | 6.6 [6.1,7.8] | 8.7 [7.7,9.8] | 6.5 [6.1,7.1] | 8.2 [7.3,9.3] |
| Parent with diabetes | Yes | 152 (85.9%) | 54 (19.4%) | 380 (91.1%) | 194 (60.8%) |
|  | No | 25 (14.1%) | 224 (80.6%) | 37 (8.9%) | 125 (39.2%) |
| Treated currently with insulin or tablets/OHA | Yes | 139 (78.5%) | 278 (100%) | 204 (48.9%) | 291 (91.2%) |
|  | No | 38 (21.5%) | 0 (100%) | 213 (51.1%) | 28 (8.8%) |

^a^ As reported previously in Shields *et al.* 2012

**Supplementary Table 2: Participant characteristics of model relevant variables for the UNITED study, used as population-representative recalibration data.** Values are displayed as median [interquartile range] for numeric variables. Categorical variables displayed as n (%).

|  |  | Early-insulin-treated (n=1,174) | | Non-early-insulin-treated (n=125) | |
| --- | --- | --- | --- | --- | --- |
|  |  | **Monogenic diabetes** | **Non-monogenic diabetes** | **Monogenic diabetes** | **Non-monogenic diabetes** |
| n |  | **10** | **1,164** | **23** | **102** |
| Gene | HNF1A | 6 (60%) | 0 (0%) | 14 (60.9%) | 0 (0%) |
|  | HNF4A | 1 (10%) | 0 (0%) | 2 (8.7%) | 0 (0%) |
|  | HNF1B | 3 (30%) | 0 (0%) | 0 (0%) | 0 (0%) |
|  | GCK | 0 (0%) | 0 (0%) | 6 (26.1%) | 0 (0%) |
|  | KCNJ11 | 0 (0%) | 0 (0%) | 1 (4.3%) | 0 (0%) |
| Age at diagnosis (years) |  | 17 [13.3,20.8] | 12 [7,19] | 20 [17.5,24.5] | 26 [22,28] |
| Age at recruitment (years) |  | 35 [28,38.8] | 28 [18,39] | 35 [25.5,42] | 34.5 [29,40.8] |
| Duration of diabetes (years) |  | 14.4 [13.7,18.1] | 14.2 [5.8,23.5] | 16.41 [2.5,22.0] | 9.5 [5.1,15.1] |
| Sex | Female | 8 (80%) | 558 (47.9%) | 11 (47.8%) | 60 (58.8%) |
|  | Male | 2 (20%) | 606 (52.1%) | 12 (52.2%) | 42 (41.2%) |
| BMI (kg/m^2^) |  | 24.2 [23.6,27.6] | 24.3 [21.3,27.6] | 27.4 [24.9,32.6] | 35.7 [31.0,42.4] |
| HbA1c (mmol/mol) |  | 63 [57,73] | 72 [63,8] | 51 [47,79] | 75 [55,91] |
| HbA1c (%) |  | 7.9 [7.4,8.8] | 8.7 [7.8,9.8] | 6.8 [6.5,9.4] | 9.0 [7.2,10.5] |
| Parent with diabetes | No | 4 (40%) | 935 (80.3%) | 3 (13.0%) | 32 (31.4%) |
|  | Yes | 6 (60%) | 229 (19.7%) | 20 (87.0%) | 70 (68.6%) |
| Treated currently with insulin or tablets/OHA | Yes | 10 (100%) | 1164 (100%) | 12 (52.2%) | 95 (93.2%) |
|  | No | 0 (0%) | 0 (0%) | 11 (47.8%) | 7 (6.9%) |
| C-peptide status/sufficient insulin production | UCPCR ≥ 0.2nmol/mmol | 10 (100%) | 240 (20.6%) |  |  |
|  | UCPCR < 0.2 nmol/mmol | 0 (0%) | 924 (79.4%) |  |  |
| Islet-antibody status | 1+ antibody positive | 0 (0%) | 162 (13.9%) |  |  |
|  | All antibodies negative | 10 (100%) | 92 (7.9%) |  |  |
|  | Not tested | 0 (0%) | 910 (78.2%) |  |  |
| Biomarker status | C-peptide negative OR any antibody positive | 0 (0%) | 1,075 (92.4%) |  |  |
|  | C-peptide positive AND All antibodies negative | 10 (100%) | 89 (7.7%) |  |  |

**Supplementary Table 3: Patient characteristics of model relevant variables from CPRD Aurum (adults of white ethnicity with a diabetes-related medcode from 01/01/2004 to 01/06/2024 diagnosed <36 years old), used as population-representative data**. Values are displayed as median [interquartile range] for numeric variables. Otherwise, categorical variables are displayed as n (%).

|  |  | Early-insulin-treated | Not-early-insulin-treated |
| --- | --- | --- | --- |
| n |  | **35,150** | **32,677** |
| Age at diagnosis (years) |  | 17 [10.7,25.5] | 31 [26.9,33.8] |
| Age at recruitment (years) |  | 42.5 [31.5,56.5] | 44.5 [37.5,53.5] |
| Duration of diabetes (years) |  | 24.2 [14.5,37] | 14.5 [7.2,23] |
| Sex | Female | 15,605 (44.4%) | 18,524 (56.7%) |
|  | Male | 19,545 (55.6%) | 14,153 (43.3%) |
| BMI (kg/m^2^) |  | 26.6 [23.4,30.4] | 33.5 [29.0,39.2] |
| HbA1c (mmol/mol) |  | 63 [54,75] | 60.5 [49,77] |
| HbA1c (%) |  | 7.9 [7.1,9.0] | 7.7 [6.6,9.2] |
| Parent with diabetes | Yes | 9,072 (25.8%) | 14,009 (42.9%) |
|  | No | 26,078 (74.2%) | 18,668 (57.1%) |
| Treated currently with insulin or tablets/OHA | Yes | 35,108 (99.9%) | 28,092 (86.0%) |
|  | No | 42 (0.1%) | 4,585 (14.0%) |

**Supplementary Table 4: Participant characteristics of model relevant variables from the external validation dataset**, composed of the UNITED paediatric study and the MY DIABETES studies. Values are displayed as median [interquartile range] for numeric variables. Otherwise, categorical variables are displayed as n (%). The monogenic diabetes column denotes grouped early-insulin-treated monogenic diabetes participants (n=5) and not-early-insulin-treated monogenic diabetes participants (n=15). ^†^UCPCR ≥0.2nmol/mmol ; unfasted plasma C-peptide <200pmol/L; fasted plasma C-peptide <80pmol/L. ^‡^ UCPCR ≥0.2nmol/mmol; unfasted plasma C-peptide ≥200pmol/L; fasted plasma C-peptide ≥80pmol/L. *Those with unknown C-peptide status are all antibody positive and therefore have a biomarker status of “C-peptide negative OR any antibody positive”. **Those with not tested islet-antibodies are all C-peptide negative and therefore have a biomarker status of “C-peptide negative OR any antibody positive”.

|  |  | Monogenic diabetes | Early-insulin-treated diabetes | Non-early-insulin-treated diabetes |
| --- | --- | --- | --- | --- |
| n |  | **20** | **935** | **70** |
| Gene | Not tested/no gene | 0 (0%) | 935 (100%) | 70 (100%) |
|  | GCK | 2 (10%) | 0 (0%) | 0 (0%) |
|  | ABCC8 | 1 (5%) | 0 (0%) | 0 (0%) |
|  | HNF1B | 2 (10%) | 0 (0%) | 0 (0%) |
|  | HNF1A | 11 (55%) | 0 (0%) | 0 (0%) |
|  | HNF4A | 4 (20%) | 0 (0%) | 0 (0%) |
| Age at diagnosis (years) |  | 14 [10.8,18.3] | 11 [6,17] | 25 [19,27] |
| Age at recruitment (years) |  | 22.9 [14,31.8] | 20.6 [13,3.6] | 33.6 [28.7,43.8] |
| Duration of diabetes (years) |  | 5.5 [1.8,22.5] | 9.3 [3.6,22.7] | 10.6 [3.6,18.4] |
| Sex | Female | 11 (55%) | 457 (48.9%) | 47 (67.1%) |
|  | Male | 9 (45%) | 478 (51.1%) | 23 (32.9%) |
| BMI (kg/m^2^) |  | 22.1 [20.2,25.7] | 23.0 [20.0,26.7] | 31.1 [25.4,34.9] |
| HbA1c (mmol/mol) |  | 60 [53,66] | 65 [56,76] | 60 [51,74] |
| HbA1c (%) |  | 7.5 [7.0,8.2] | 8.1 [7.3,9.1] | 7.6 [6.9,8.9] |
| Parent with diabetes | No | 2 (10%) | 755 (80.8%) | 31 (44.3%) |
|  | Yes | 18 (90%) | 180 (19.2%) | 39 (55.7%) |
| Treated currently with insulin or tablets/OHA | Yes | 16 (80%) | 933 (99.8%) | 65 (92.9%) |
|  | No | 4 (20%) | 0 (0%) | 5 (7.1%) |
|  | Unknown | 0 (0%) | 2 (0.21%) | 0 (0%) |
| C-peptide status/sufficient insulin production | Sufficient^†^ | 20 (100%) | 184 (19.7%) | 55 (78.6%) |
|  | Insufficient^‡^ | 0 (0%) | 745 (79.7%) | 14 (20%) |
|  | Unknown | 0 (0%) | 6 (0.6%) * | 1 (1.4%) |
| Islet-antibody status | 1+ antibody positive | 0 (0%) | 441 (47.2%) | 19 (27.1%) |
|  | All antibodies negative | 20 (100%) | 247 (26.4%) | 51 (72.9%) |
|  | Not tested | 0 (0%) | 247 (26.4%)** | 0 (0%) |
| Biomarker status | C-peptide negative OR any antibody positive | 0 (0%) | 891 (95.3%) | 27 (38.6%) |
|  | C-peptide positive AND All antibodies negative | 20 (100%) | 44 (4.7%) | 42 (60%) |
|  | Antibody negative & unknown C-peptide | 0 (0%) | 0 (0%) | 1 (1.4%) |

**Supplementary Figure 7: Calibration plots in UNITED recalibration data for A) early-insulin-treated prediction model (clinical features, with biomarkers) and B) not-early-insulin-treated model.** The population is stratified into participants tested for a monogenic diabetes gene (triangles), or participants not tested for a monogenic diabetes gene due to being islet autoantibody positive or C-peptide negative (squares). Each group is split into tertiles according to the predicted monogenic diabetes probabilities form the model, due to the small number of monogenic diabetes cases in each quantile, and then the y-axis denotes the proportion of monogenic diabetes cases found in each tertile in the raw data, and the x-axis is the median *predicted* monogenic diabetes probability within each tertile. Error bars denote confidence intervals of the observed probability.

**Supplementary Figure 8: Receiver Operating Characteristic curve for the discriminatory ability of biomarker status (1 if C-peptide negative or 1+ islet autoantibody positive, n=1,075; 0 if C-peptide positive and no positive islet autoantibodies, n=99) for predicting monogenic diabetes status in early-insulin-treated participants of UNITED (n=1,174, monogenic diabetes = 10).** Text represents the Area Under the Curve (AUC) and 95% confidence interval computed from 2000 stratified bootstrap replicates. Sensitivity for predicting monogenic diabetes = 100% (for biomarker status = 0), specificity for monogenic diabetes = 92.35%

**Supplementary Figure 9: Distribution of monogenic diabetes model probabilities in UNITED** for A) early-insulin-treated participants using A.1) clinical features alone (mean monogenic diabetes probability: monogenic diabetes = 2.1%; non-monogenic diabetes = 0.9%) and A.2) clinical features and biomarkers (mean monogenic diabetes probability: monogenic diabetes = 14.0%; non-monogenic diabetes = 0.8%); and B) not-early-insulin-treated participants of UNITED (mean monogenic diabetes probability: monogenic diabetes = 44.5%; non-monogenic diabetes = 12.5%).

**Supplementary Figure 10: Density plot of predicted monogenic diabetes probabilities for 67,827 adult CPRD individuals** of white ethnicity, diagnosed under or equal the age of 35, with a diabetes-related medcode between 01/01/2004 and 01/06/2024, that are registered with a GP on 01/01/2024. The clinical features only model was used for early-insulin-treated individuals. The solid black line represents the estimated monogenic diabetes prevalence of 3.6% (Shields *et al.* 2017), and the dashed line represents the mean monogenic diabetes probability found in this population-representative cohort of 3.8%.

**Supplementary Table 5: Assessment of extended potential testing thresholds of the monogenic diabetes Calculator in the combined UNITED dataset.** For early-insulin-treated participants, the clinical and biomarker model was used. The first column denotes the examined monogenic diabetes probability threshold. The second column denotes the % of participants (of the total) who have a monogenic diabetes probability greater than the respective threshold and would therefore be theoretically eligible for monogenic diabetes testing. The third column describes the percentage of monogenic diabetes cases (of the total monogenic diabetes cases) that would be missed (i.e. have a monogenic diabetes probability below the examined threshold) at this threshold. The fourth column states the positive test rate (or positive predictive value = PPV) within those eligible for monogenic diabetes testing (based on their monogenic diabetes probability being greater than the examined testing threshold. The fifth column denotes the negative predictive value (NPV), which is the percentage of those who are non-monogenic diabetes, out of those who are under the respective threshold and therefore correctly not eligible for monogenic diabetes genetic testing. The final two columns are sensitivity (the proportion of monogenic diabetes cases that have a monogenic diabetes probability greater than the threshold, of the total monogenic diabetes cases, and therefore eligible for monogenic diabetes testing) and specificity (the proportion of non-monogenic diabetes cases that have a monogenic diabetes probability less than the threshold, of the total non-monogenic diabetes cases, and therefore not eligible for monogenic diabetes genetic testing).

| UNITED (n=1,299; 33 monogenic diabetes cases within this group) | | | | | | |
| --- | --- | --- | --- | --- | --- | --- |
| **Threshold** | **% individuals tested at threshold** | **% monogenic diabetes cases missed** | **% positive test rate (PPV)** | **% negative test rate (NPV)** | **Sensitivity** | **Specificity** |
| ≥5% | 12.5%  (163/1,299) | 6.1%  (2/33) | 19.0%  (31/163) | 99.8%  (1,134/1,136) | 93.9%  (31/33) | 89.6%  (1,134/1,266) |
| ≥10% | 7.3%  (95/1,299) | 15.2%  (5/33) | 29.5%  (28/95) | 99.6%  (1,199/1,204) | 84.8%  (28/33) | 94.7%  (1,199/1,266) |
| ≥15% | 4.8%  (62/1,299) | 27.3%  (9/33) | 38.7%  (24/62) | 99.3%  (1,228/1,237) | 72.7%  (24/33) | 97.0%  (1,228/1,266) |
| ≥20% | 3.4%  (44/1,299) | 39.4%  (13/33) | 45.5%  (20/44) | 99.0%  (1,209/1,255) | 60.6%  (20/33) | 98.1%  (1,209/1,266) |
| ≥25% | 3.0%  (39/1,299) | 45.5%  (15/33) | 46.2%  (18/39) | 98.8%  (1,242/1,260) | 54.5%  (18/33) | 98.3%  (1,242/1,266) |
| ≥30% | 2.2%  (28/1,299) | 60.6%  (20/33) | 46.4%  (13/28) | 98.4%  (1,251/1,271) | 39.4%  (13/33) | 98.8%  (1,251/1,266) |

**Supplementary Table 6: Comparison of monogenic diabetes calculator to alternative strategies in combined UNITED recalibration data.** *Biomarker screening pathway (20): Testing criteria = UCPCR ≥0.2nmol/mmol and GAD and IA2 islet autoantibody negative (if early-insulin-treated) or GAD and IA2 negative (if not-early-insulin-treated). **Traditional monogenic diabetes criteria (6): Testing criteria = age at diagnosis <25 years, not-early-insulin-treated, and at least one parent affected by diabetes. †Testing criteria = ≥5% probability on original MODY calculator, irrespective of biomarker status. ‡Testing criteria = C-peptide positive, islet autoantibody negative and ≥5% probability on original MODY calculator (if early-insulin-treated) or ≥5% probability on original MODY calculator (if not-early-insulin-treated). #Testing criteria = C-peptide positive, islet autoantibody negative and ≥10% probability on original MODY calculator (if early-insulin-treated); or ≥20% on original MODY calculator (if early-insulin-treated) (18).

| UNITED (n=1,299; 33 monogenic diabetes cases within this group) | | | | | | |
| --- | --- | --- | --- | --- | --- | --- |
| **Threshold** | **% individuals tested at threshold** | **% monogenic diabetes cases missed** | **% positive test rate (PPV)** | **% negative test rate (NPV)** | **Sensitivity** | **Specificity** |
| Monogenic diabetes Probability Calculator (≥5%) | 12.5%  (163/1,299) | 6.1%  (2/33) | 19.0%  (31/163) | 99.8%  (1,134/1,136) | 93.9%  (31/33) | 89.6%  (1,134/1,266) |
| Biomarker screening pathway* | 17.2%  (223/1,299) | 0%  (0/33) | 14.8%  (33/223) | 100%  (1,076/1,076) | 100%  (33/33) | 85.0%  (1,076/1,266) |
| Traditional monogenic diabetes criteria** | 2.8%  (37/1,299) | 54.5%  (18/33) | 40.5%  (15/37) | 98.6%  (1,244/1,261) | 45.5%  (15/33) | 98.3%  (1,244/1,266) |
| Original MODY Probability Calculator (≥5%)† | 57.1%  (742/1,299) | 9.1%  (3/33) | 4%  (30/742) | 99.5%  (554/557) | 90.9%  (30/33) | 43.8%  (554/1,266) |
| Original MODY Probability Calculator after biomarker-based triaging ‡ | 11.5%  (149/1,299) | 9.1%  (3/33) | 20.1%  (30/149) | 99.7%  (1,147/1,150) | 90.9%  (30/33) | 90.6% (1,147/1,266) |
| Current NHS genomic testing criteria# | 8.7%  (113/1,299) | 12.1%  (4/33) | 25.7%  (29/113) | 99.7%  (1,182/1,186) | 87.9%  (29/33) | 93.4% (1,182/1,266) |

**Supplementary Table 7:** **Assessment of extended potential testing thresholds of the Monogenic Diabetes Calculator in the combined external validation dataset.** For early-insulin-treated participants, the clinical and biomarker model was used. The first column denotes the examined monogenic diabetes probability threshold. The second column denotes the % of participants (of the total) who have a monogenic diabetes probability greater than the respective threshold and would therefore be theoretically eligible for monogenic diabetes testing. The third column describes the percentage of cases (of the total monogenic diabetes cases) that would be missed (i.e. have a monogenic diabetes probability below the examined threshold) at this threshold. The fourth column states the positive test rate (or positive predictive value = PPV) within those eligible for monogenic diabetes testing (based on their monogenic diabetes probability being greater than the examined testing threshold. The fifth column denotes the negative predictive value (NPV), which is the percentage of those who are non-monogenic diabetes, out of those who are under the respective threshold and therefore correctly not eligible for monogenic diabetes genetic testing. The final two columns are sensitivity (the proportion of monogenic diabetes cases that have a monogenic diabetes probability greater than the threshold, of the total monogenic diabetes cases, and therefore eligible for monogenic diabetes testing) and specificity (the proportion of non-monogenic diabetes cases that have a monogenic diabetes probability less than the threshold, of the total non-monogenic diabetes cases, and therefore not eligible for monogenic diabetes genetic testing).

| External validation cohort (n=1,025; 20 monogenic diabetes cases within this group) | | | | | | |
| --- | --- | --- | --- | --- | --- | --- |
| **Threshold** | **% individuals tested at threshold** | **% monogenic diabetes cases missed** | **% positive test rate (PPV)** | **% negative test rate (NPV)** | **Sensitivity** | **Specificity** |
| ≥5% | 12.1%  (n=124/1,025) | 0.0%  (n=0/20) | 16.1%  (n=20/124) | 100%  (n=901/901) | 100%  (n=20/20) | 89.7%  (n=901/1,005) |
| ≥10% | 9.0%  (n=92/1,025) | 10.0%  (n=2/20) | 19.6%  (n=18/92) | 99.8%  (n=931/933) | 90.0%  (n=18/20) | 92.6%  (n=931/1,005) |
| ≥15% | 6.5%  (n=67/1,025) | 10.0%  (n=2/20) | 26.9%  (n=18/67) | 99.8%  (n=956/958) | 90.0%  (n=18/20) | 95.1%  (n=956/1,005) |
| ≥20% | 5.4%  (n=55/1,025) | 15.0%  (n=3/20) | 30.9%  (n=17/55) | 99.7%  (n=967/970) | 85.0%  (n=17/20) | 96.2%  (n=967/1,005) |
| ≥25% | 4.0%  (n=41/1,025) | 30.0%  (n=6/20) | 34.1%  (n=14/41) | 99.4%  (n=978/984) | 70.0%  (n=14/20) | 97.3%  (n=978/1,005) |
| ≥30% | 3.2%  (n=33/1,025) | 30.0%  (n=6/20) | 42.4%  (n=14/33) | 99.4%  (n=986/992) | 70.0%  (n=14/20) | 98.1%  (n=986/1,005) |

**Supplementary Figure 11: Calibration plots for A) early-insulin-treated prediction model (clinical features, with biomarkers model) and B) not-early-insulin-treated model in the external validation dataset.** The population is stratified into patients tested for a monogenic diabetes gene (triangles), or patients not tested for a monogenic diabetes gene due to being islet autoantibody positive or C-peptide negative (squares). Each group is split into tertiles according to the predicted monogenic diabetes probabilities form the model, due to the small number of monogenic diabetes cases in each quantile, and then the y-axis denotes the proportion of monogenic diabetes cases found in each tertile in the raw data, and the x-axis is the median *predicted* monogenic diabetes probability within each tertile. Error bars denote confidence intervals of the observed probability.

**Supplementary Table 8: Assessment of using a ≥5% probability threshold to prompt genetic testing in a paediatric subgroup of the combined external dataset.**

| Threshold | % individuals tested at threshold | % monogenic diabetes cases missed | % positive test rate (PPV) | % negative test rate (NPV) | Sensitivity | Specificity |
| --- | --- | --- | --- | --- | --- | --- |
| Diagnosed < 18 years | 3.5% (25/718) | 0% (0/3) | 12% (3/25) | 100% (693/693) | 100% (3/3) | 96.9% (693/715) |
| Diagnosed & recruited < 18 years | 5.0% (22/438) | 0% (0/2) | 9.1% (2/22) | 100% (416/416) | 100% (2/2) | 95.4% (416/436) |

**Supplementary Table 9: Model parameter estimates for the early- and not-early-insulin-treated models, per unit change.** The model parameters provided for early-insulin-treated individuals are only applicable when all biomarkers have been measured, and they are: C-peptide ≥200pmol/L AND no islet autoantibodies present. The number in parentheses correspond to the 95% credible intervals. Model form is: $\log\left( \frac{{p_{i}}^{U}}{1-{p_{i}}^{U}} \right)= \gamma_{0}+\gamma_{1}z_{i}$, where the left-hand side of the equation denotes the log-odds, and $z_{i}$denotes the linear predictor for each individual *i* (linear predictor = the linear model equation made of model parameter estimates below).

| Model parameters | Early-insulin-treated Model | Not-early-insulin-treated Model |
| --- | --- | --- |
| Intercept | 1.57 (-0.61; 3.84) | 18.66 (15.17; 22.55) |
| At least one parent affected with diabetes | 3.22 (2.56; 3.93) | 2.01 (1.22; 2.83) |
| Age at recruitment (years) | -0.08 (-0.11; -0.06) | -0.03 (-0.06; -0.004) |
| HbA_1c_ (%) | -0.66 (-0.88; -0.47) | -0.58 (-0.82; -0.36) |
| Age at diagnosis (years) | 0.10 (0.06; 0.15) | -0.31 (-0.38; -0.25) |
| Sex (baseline Male) | 1.44 (0.75; 2.14) | 0.66 (0.03; 1.30) |
| Currently treated with insulin or tablets | - | -1.28 (-2.14; -0.45) |
| BMI (kg/m^2^) | - | -0.23 (-0.29; -0.17) |
| Gamma 0 ($\boldsymbol{\gamma}_{\boldsymbol{0}}\boldsymbol{)}$ | -1.84 (-2.64; -1.12) | -1.45 (-2.10; -0.85) |
| Gamma 1 $\boldsymbol{(\gamma}_{\boldsymbol{1}}\boldsymbol{)}$ | 0.36 (-0.001; 0.76) | 0.47 (0.28; 0.72) |
